# The Effect of a Two-Stage Heat-Treatment on the Microstructural and Mechanical Properties of a Maraging Steel

**DOI:** 10.3390/ma10121346

**Published:** 2017-11-23

**Authors:** Thomas Hadfield Simm, Lin Sun, Deri Rhys Galvin, Paul Hill, Martin Rawson, Soran Birosca, Elliot Paul Gilbert, Harshad Bhadeshia, Karen Perkins

**Affiliations:** 1 College of Engineering, Swansea University, Swansea SA1 8EN, UK; derigalvin@gmail.com (D.R.G.); S.Birosca@Swansea.ac.uk (S.B.); k.m.perkins@swansea.ac.uk (K.P.); 2 Department of Materials Science and Metallurgy, University of Cambridge, Cambridge CB3 0FS, UK; permax@gmail.com (L.S.); hkdb@cam.ac.uk (H.B.); 3 Rolls-Royce plc, P.O. Box 31, Derby DE24 8BJ, UK; Paul.Hill2@Rolls-Royce.com (P.H.); Martin.Rawson@Rolls-Royce.com (M.R.); 4 The Australian Nuclear Science and Technology Organisation (ANSTO), Lucas Heights NSW 2234, Australia; epg@ansto.gov.au

**Keywords:** small angle neutron scattering, maraging steel, precipitation strengthening, NiAl, Laves phase, atom probe tomography, creep, strength, ductile to brittle transition temperature

## Abstract

Maraging steels gain many of their beneficial properties from heat treatments which induce the precipitation of intermetallic compounds. We consider here a two-stage heat-treatment, first involving austenitisation, followed by quenching to produce martensite and then an ageing treatment at a lower temperature to precipitation harden the martensite of a maraging steel. It is shown that with a suitable choice of the initial austenitisation temperature, the steel can be heat treated to produce enhanced toughness, strength and creep resistance. A combination of small angle neutron scattering, scanning electron microscopy, electron back-scattered diffraction, and atom probe tomography were used to relate the microstructural changes to mechanical properties. It is shown that such a combination of characterisation methods is necessary to quantify this complex alloy, and relate these microstructural changes to mechanical properties. It is concluded that a higher austenitisation temperature leads to a greater volume fraction of smaller Laves phase precipitates formed during ageing, which increase the strength and creep resistance but reduces toughness.

## 1. Introduction

Maraging steels are used in a range of industrial sectors, most notably for aerospace applications. They gain their beneficial properties from their martensitic microstructure containing a dispersion of small precipitates. In this work, we consider two types of precipitates: Laves phase and NiAl. Laves phase [(Fe, Cr)_2_ (Mo, W)] [1] is found in a range of steel alloy systems with high amounts of Cr or Mo, such as stainless or maraging steels [2,3,4,5]. It is generally believed to improve the strength at the expense of toughness [4,5,6]. The contribution to creep behaviour is more complex; the resistance to creep can increase via strengthening by precipitation strengthening [1,7], but the resulting loss of solute can reduce the solid solution strengthening component, particularly during extended service at elevated temperatures [2,3,4]. In alloys similar to the one studied here [8,9], Laves phase had a tendency to form on boundaries and can range in size between around 10 nm and several 100 nm.

NiAl [1] is an intermetallic phase which can form in steels with sufficient quantities of nickel and aluminium, and low concentrations of titanium [10,11,12,13]. It has a B2 superlattice crystal structure with a lattice constant close to that of ferrite [14,15], and hence can remain coherent with the matrix over long ageing times [16]. It therefore is one of the most effective strengtheners [13], but can lead to poor ductility and toughness [17] while enhancing creep resistance [11] at temperatures below 600 °C. However, the necessary nickel can also compromise creep strength if it stimulates the formation of reverted austenite [17,18].

In previous work on this alloy, the influence of martensitic microstructure have been studied [19], and the phases and precipitation in the alloy have been studied by transmission electron microscopy (TEM) and atom probe tomography (APT) [8]. In a previous work a model [9] was presented to relate the precipitate volume fraction and size from small angle neutron scattering (SANS) measurements with the strength of three similar alloys one of which (F1E) is the one studied in this work. The components of strengthening were separated into: (a) precipitation strengthening due to Laves (Δ*σ_L_*) and NiAl (Δ*σ_β_*); (b) solid solution strengthening (Δ*σ_SS_*); and (c) strengthening (Δ*σ_mart_*) due to the structure the lath martensite and the intrinsic strength of iron (which can be considered as the stress needed to move a dislocation in a perfect BCC crystal structure, i.e., with no grain boundary or solid solution strengthening). In the present work, this model is used to investigate the effect of a two-stage heat-treatment. We consider the effect of austenitisation at three different temperatures followed by an ageing treatment to stimulate precipitation in the martensite that forms after cooling from the austenite phase field. Multiple precipitate ageing treatments are a common feature of processing [20,21] they introduce variety in the precipitate population and can induce the formation of additional phases. It is demonstrated how such a two-stage treatment can have dramatic effect on the mechanical properties of an alloy, data that are explained on the basis of detailed microstructural characterisation.

## 2. Materials and Methods

The composition of the alloy is shown in Table 1. After production, homogenisation and forging, the alloy was austenitised at three different temperatures: 825 °C for 2 h, 870 °C for 1 h, 960 °C for 1 h; and then water quenched. It was subsequently aged at 540 °C for a range of times up to 24 h. Samples aged for 7.5 h were used for subsequent tensile and creep tests, whilst smaller samples aged for a range of times were used for microstructural and hardness measurements.

Mechanical testing was carried out at Swansea Materials Research & Testing Ltd (SMaRT), Swansea University, Singleton and Bay Campus, Swansea, UK. Tensile tests were conducted at a strain-rate of 0.00010 s^−1^ using the standard BS EN ISO 6892-1: 2016. Creep testing was carried out by applying a constant stress to the sample, of 0.65 of the elevated yield strength (at 0.2% strain), at a temperature of 500 °C following the standard BS EN 10291:2000. Both creep and tensile test pieces have the same dimensions with a gauge length of 20 mm and diameter 4 mm. Hardness measurements were made with a load of 20 kg using a Vickers hardness testing machine from polished samples.

Atom probe tomography (APT) specimens were analysed using a Cameca LEAP 3000X HR atom probe, at Oxford University; with reconstruction and analysis being conducted using IVAS version 3.6.6 (details elsewhere [8,22]).

Scanning electron microscopy (SEM) was done using a Hitachi SU3500 (Hitachi, Tokyo, Japan) with a tungsten filament at Swansea University. Electron back-scattered diffraction (EBSD) orientation maps were determined using a voltage of 20 kV, and a step size of 0.7 μm. SEM imaging was done using a back-scattered detector and a voltage of 15 kV with a spot size of 60 (emission current of ~0.1 A), and a working distance of ~5 mm. These back-scattered images were used to determine size and area fractions of precipitates; analysis was done using Image-J from at least 4 maps at 3500× magnification. Laves phase appears slightly diffuse and there is a limitation on the size that can accurately be determined, hence regions of higher contrast with area less than 0.008 μm^2^ are excluded from precipitate analysis.

Small angle neutron scattering (SANS) experiments were conducted on the Quokka instrument at the OPAL reactor at ANSTO, Sydney, Australia [23]. Three configurations were used to cover a *q* range of ~0.003 to 0.74 Å^−1^ where *q* is the magnitude of the scattering vector defined as *q* = 4*π*/*λ·sinθ*, where *λ* = 5 Å with Δ*λ*/*λ* = 10% resolution and 2*θ* the scattering angle. These configurations were *L1 = L2 =* 20 m, *L1 = L2 =* 12 m, and *L1 =* 12 m, *L2 =* 1.3 m, where *L1* and *L2* are source-to-sample and sample-to-detector distances, and with source and sample apertures of 50 mm and 5 mm diameter respectively. Analysis was carried out using SasView [24]. The background and matrix scattering was modelled using a Guinier Porod function; the Laves and NiAl phases were separated based on their size, since it is expected that NiAl will be smaller at all stages. More details of the SANS experimental set-up and analysis procedure can be found in the appendix and in a separate work [9]. Laves phase is separated into two types: (1) austenitisation Laves, formed during austenitisation; and (2) ageing Laves, formed during lower temperature ageing in the martensite region. Above the highest austenitisation temperature (960 °C) it is assumed no austenitisation Laves phase form. This is consistent with thermodynamics calculations using MatCalc using the Fe 2.009 database, and SEM and TEM measurements after austenitisation. NiAl and ageing Laves phase are both modelled as spherical in shape, and austenitisation Laves phase as an ellipsoid; the spherical shape assumed for ageing Laves (previous work [9] used an ellipse) is due the difficulty of fitting to all three phases.

## 3. Results

### 3.1. Martensite Microstructure

**Figure 1:**
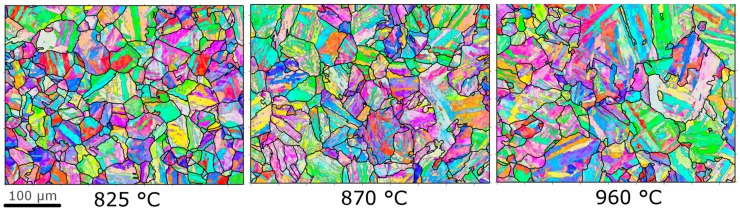
EBSD (Electron back-scattered diffraction) maps of the martensitic microstructure after different austenitisation conditions prior to ageing. Black lines indicate PAG (Prior austenite grain) reconstructions (using [26]) and grey lines are boundaries with misorientation greater than 5°.

EBSD maps showing the martensitic microstructure after the austenitisation treatments are given in Figure 1. The data were analysed and plotted using MTEX [25], with the data cleaned using a spline filter. Prior austenite grain (PAG) reconstruction was performed using the work of Nyyssonen [26]. The microstructure is typical of low carbon steels with prior austenite grains, which can be subdivided into blocks (regions of the same martensitic variant) separated by misorientations more than ~10°, and then laths of the same crystallographic variant, with a small misorientation across them [27,28]. The highest austenitisation temperature has larger PAG sizes, as determined by a linear intercept method using PAG reconstruction [26]: 40.5 μm for 960 °C, 29.8 μm for 870 °C, and 27.4 μm for 825 °C. With standard deviations of 24.4, 8.9 and 7.6 μm. The standard deviation was calculated using Equation (14) from ASTM E112-12: the standard deviation (s) of a variable *x*, with individual values of *x_i_* (*i* = 1 to *n*) and mean $\bar{x}$, is given by s=[∑(xi−x¯)2n−1]12.

The larger PAG size is probably the result of grain growth during austenitisation, since this will be greater at the higher austenitisation temperatures (even though the austenitisation time is lower for 960 °C). From EBSD measurements, it is found that there is also a difference in the morphology of martensite produced by the different heat-treatments. The block sizes are determined from the linear intercept method for boundaries of misorientations greater than 15°: 11.2 μm for 960 °C, 9.0 μm for 870 °C, and 10.1 μm for 825 °C. With standard deviations of 1.3, 0.7 and 1.1 μm respectively. The lowest austenitisation treatment has more block boundaries than for the highest austenitisation treatment. This is probably due to the smaller PAG size, since a relationship between block and PAG sizes has been found in previous work [19]. The precipitates formed during austenitisation could act as nucleation points for martensite or may stop the progress of martensite as it forms, but there is no clear evidence for this based on these maps.

### 3.2. Precipitation

#### 3.2.1. SEM and APT

**Figure 2:**
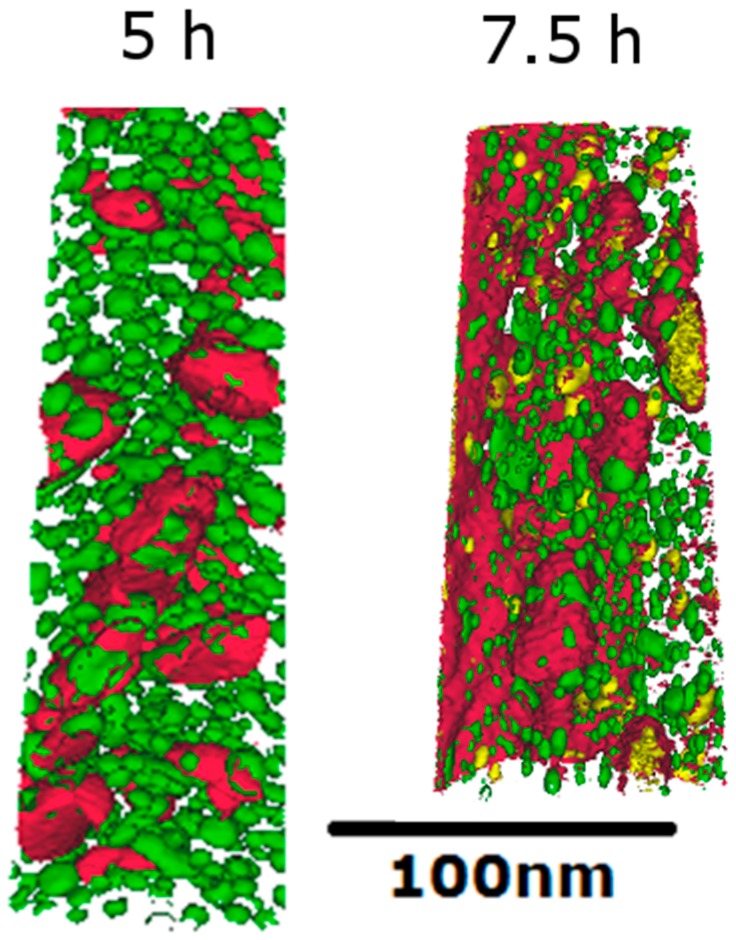
Isosurfaces from APT (Atom probe tomography) of the alloys, of Ni in green to represent NiAl, Mo in red and Cr in yellow to represent Laves phase. The image on the left is after ageing for 5 h and on the right after ageing for 7.5 h; both are after austenitisation at 960 °C.

**Figure 3:**
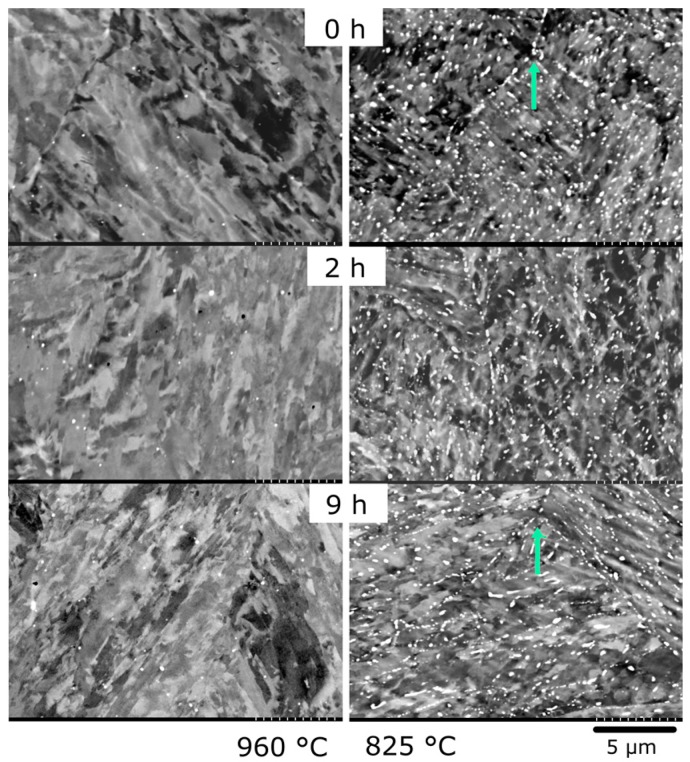
Back-scattered SEM (Scanning electron microscopy) images of the high and low austenitisation treatments after austenitisation and ageing at a range of times. The light spots represent Laves phase precipitates, and the arrows represent probable PAG triple points.

The alloy consists of two main precipitates: Laves phase (rich in Mo, W and Cr) and NiAl precipitates (rich in Ni and Al). The precipitation in this alloy is discussed in more detail, based on a more detailed TEM and APT examination, in a separate work [8] and some of the comments below are based on this work. As shown in Figure 2, after ageing, the NiAl phase is small (around 4 nm), almost spherical and distributed evenly throughout the matrix. In contrast, the Laves phase (Figure 2 and Figure 3) are more irregular in shape, display a tendency to form on laths or other grain boundaries, and are generally larger in size.

**Figure 4:**
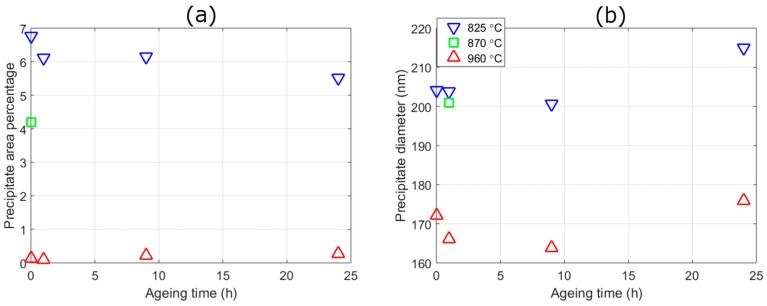
Precipitate area percentage (**a**) and precipitate equivalent circular diameter (**b**) from back-scattered SEM images and image-J analysis. The three different austenitisation temperatures are shown at a range of ageing times (870 °C has only been measured at 0 h). An indication of the errors in the results are shown in Table 2.

During the lower austenitisation temperatures (825 °C and 870 °C) large Laves phase (~200 nm) form (Table 2 and Figure 3 and Figure 4), which we shall refer to as “austenitisation Laves phase”. Previous work [8] using SEM, TEM and APT showed that small Laves phase or NiAl do not exist after austenitisation temperatures of 825 °C or above. At the highest austenitisation temperature (960 °C) there are only a few of these austenitisation Laves phase precipitates (area fraction from SEM of ~0.1%), whereas at the lowest temperature (825 °C) there are many more precipitates (area fraction ~6%), with an area fraction between the two at 870 °C (~4%). This austenitisation Laves phase, has a tendency to form on austenite grain boundaries (possible prior austenite grain (PAG) boundaries are highlighted in Figure 3), but they can also form within the austenite grains. During ageing the volume fraction and size of these austenitisation Laves phase remain approximately the same; however, since only Laves phase with an area greater than 0.008 μm^2^ can be determined accurately from these back scattered images, it cannot be used to understand Laves formed during ageing, or NiAl. The APT maps in Figure 2 provide more details of these ageing Laves phase precipitates; from these measurements (and from TEM) we find that they are ~20 nm in size after ageing for 5 h at 540 °C with an almost ellipsoidal shape.

#### 3.2.2. SANS

**Figure 5:**
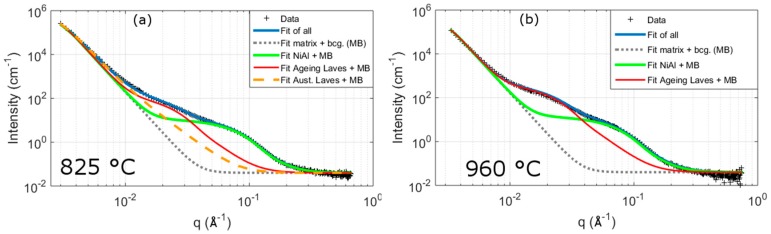
Example fits to the SANS (Small angle neutron scatterin) data after ageing at 540 °C for 7.5 h. Fits to the data with (**a**): the an initial 825 °C austenitisation treatment and (**b**): after austenitisation at 960 °C. The fits to the different components are added to the matrix plus background contribution (MB).

To quantify the precipitate population during the heat-treatments, SANS measurements were conducted. The advantage of this technique is that it can be used to quantify size and volume fractions of precipitates for a much larger volume than by TEM or APT, hence yielding a bulk average along with the ability to measure many samples in a single experiment. However, there are difficulties with the interpretation of results because they are indirect and require additional assumptions. Example SANS data and simulated profiles for the three phases are shown in Figure 5. As with previous work [9] a number of assumptions are made, these include: (1) precipitates are separated based on their size (i.e., in order of decreasing size: austenitisation Laves, then ageing Laves, then NiAl); (2) the scattering from the martensitic matrix and background is assumed to be constant; (3) the size distribution of each precipitate type is assumed to be constant (i.e., a constant ratio of standard deviation to mean size, and remain as either spheres or ellipsoids); (4) the scattering length density of Laves phase and NiAl are determined so that the SANS volume fraction values match those from APT (this is due to difficulties in obtaining a reliable volume fraction from the expected compositions from APT, see the Appendix A); (5) ageing and austenitisation Laves phase precipitates are assumed to have the same composition and scattering length densities.

**Figure 6:**
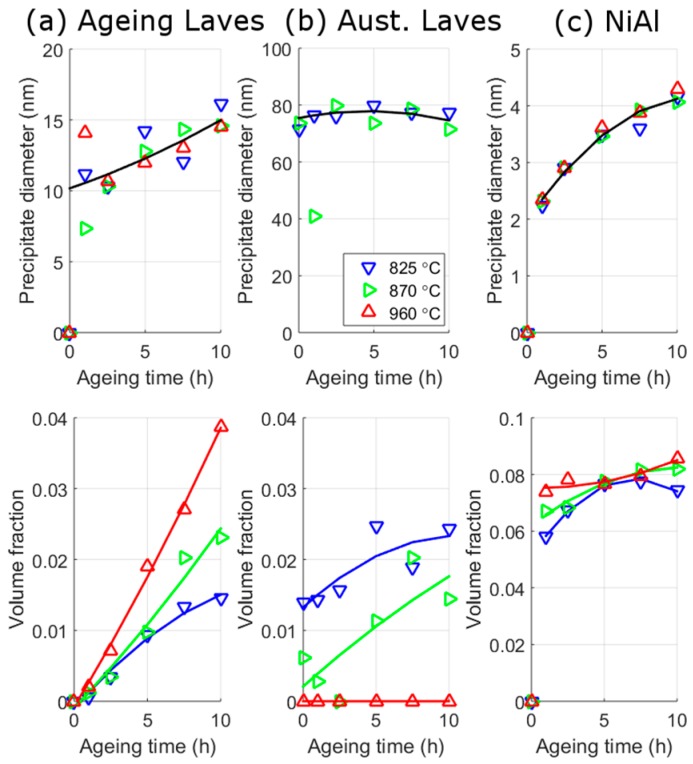
Results from SANS analysis of the change in size of precipitates (**top**) and the volume fraction of precipitates (**bottom**), with different ageing times at 540 °C after different austenitisation temperatures. Laves phase are separated based on whether they are formed during ageing (**a**) or austenitisation (**b**), and NiAl is ageing only (**c**).

Figure 6 illustrates the changes in precipitate size and volume fraction of NiAl and Laves phase after ageing at 540 °C. The precipitates are separated based on their size with the order in decreasing sizes at all ageing conditions based on other measurements of: NiAl, then ageing Laves phase, and then austenitisation Laves phase. Figure 6 shows that the size of both NiAl and ageing Laves phase increase with ageing time following a similar power law relationship. This is expected from the theory of precipitate evolution [29]. The size of austenitisation Laves phase precipitates are approximately ten times larger than the other precipitates (with a diameter of ~90 nm); and their size remains approximately constant with ageing. There is a greater uncertainty in the austenitisation Laves phase than the other precipitates due to the limited range of *q* used, which cuts off part of the austenitisation Laves phase’s profile, and the overlap of the other precipitates profiles (Figure 5). Hence, the difference in size between SANS and SEM could be due to the underlying assumptions involved in the SANS models. An interesting feature of the results in Figure 6 is that the size of ageing and austenitisation Laves phase and NiAl, are approximately the same for the three austenitisation temperatures at a given ageing time.

The SANS results (Figure 6) show that the two precipitates are in different stages of their evolution during the times considered. Both types of Laves phase increase in volume fraction across all temperatures and times measured, which is typical of a precipitate in the growth stage; whereas NiAl maintains an almost constant volume fraction with ageing time, which is typical of a precipitate in the coarsening stage. In a similar manner to their size values, the volume fraction of NiAl are also approximately the same after the different austenitisation treatments. There appears to be a slight increase in the volume fraction of NiAl with increased austenitisation temperature, which is particularly noticeable at the two lowest measured times (1 h and 2.5 h). This may be indicative of austenitisation Laves phase influencing the formation of the ageing precipitates. However, there is also the possibility that it may be an artefact of fitting, further work would be needed to determine which of these is the case. More ageing Laves phase will be present with increased austenitisation temperatures (Figure 6c), and some of the NiAl profile may be due to scattering contributions from the smaller ageing Laves phase precipitates. The size of austenitisation Laves are constant with ageing times and have magnitudes that are less than half of those found by SEM. This difference in size from the two techniques may be a result of what size constitutes for each method, but is more likely a result of the difficulty in determining austenitisation Laves phase due to the high amount of matrix scattering and the limited range of *q*-values measured at. The main difference between the austenitisation treatments are given by the relative volume fractions of ageing and austenitisation Laves phase. The greater the austenitisation temperature, the greater is the volume fraction of ageing Laves phase and the smaller is the volume fraction of austenitisation Laves phase. The volume fraction of ageing Laves phase is approximately two times larger for the 960 °C treatment than the 825 °C treatment. In contrast, for the 825 °C treatment, which has the highest volume fraction of austenitisation Laves phase, the volume fraction is higher for the austenitisation Laves phase than the ageing Laves phase. There is no austenitisation Laves phase present for the 960 °C treatment because it was not set as a fitting variable, although as shown in the SEM results there are some Laves phase present from previous processing these do not change in area fraction with ageing, whereas 870 °C has approximately half the volume fraction of 825 °C. The volume fraction of austenitisation Laves phase is consistent with the analysis from the SEM images (Figure 4), in that they are relatively constant for both. It should be noted that area fractions determined from SEM are different from volume fraction, since in the former an account of the interaction depth of the backscattered electrons must be made to convert to the latter.

**Figure 7:**
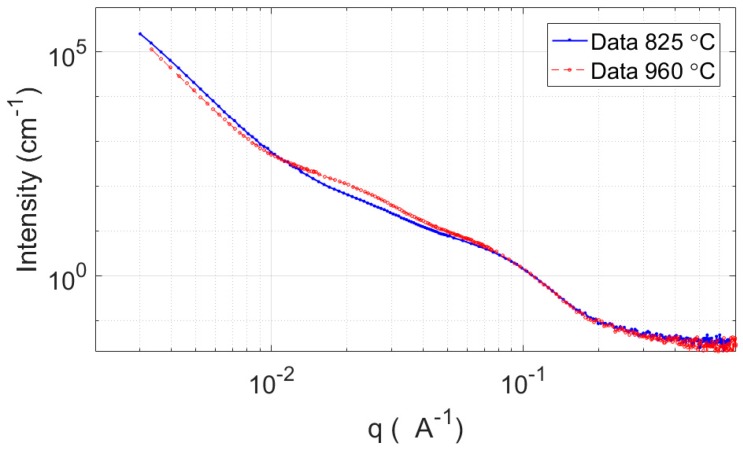
SANS data after ageing at 540 °C for 7.5 h for austenitisation conditions of 825 °C and 960 °C.

The difference in the volume fractions of the two types of Laves phase can be seen from the SANS data after ageing for 7.5 h in Figure 7. In the case shown here, and all other ageing times measured, there is a greater intensity at low *q*-values, which correspond to larger precipitates, for the low austenitisation treatment compared to the higher austenitisation treatments at a given ageing time. Whilst the intensity at *q* ~ 0.02 Å^−1^ is always higher for the higher austenitisation treatments. This is consistent with the analysis presented above, of an increase in smaller ageing Laves (*q* ~ 0.02 Å^−1^) and a decrease in austenitisation Laves (low *q*) with increasing austenitisation temperature.

As described in a previous work [9], the volume fraction and size of ageing Laves phase and NiAl are consistent with TEM and APT results. It is worth repeating that the SANS volume fraction values are calculated from an arbitrary scattering length density, rather than one calculated based on composition from other measurements (scattering length density is calculated from the composition and density of phases); i.e., the volume fractions are adjusted by a constant factor to give results comparable to other techniques. This was done because volume fraction values using scattering length densities calculated from APT composition measurements deviated significantly from other measurements; this is discussed in more detail in the Appendix and [9]. From APT measurements of the alloy after austenitisation at 825 °C, the volume fraction of austenitisation Laves phase was calculated to be 0.95% using the composition of precipitate, matrix and starting composition using the Lever rule method. This volume fraction, albeit from one APT sample containing one austenitisation Laves phase precipitate, is consistent with the SANS results shown.

### 3.3. Mechanical Tests

**Figure 8:**
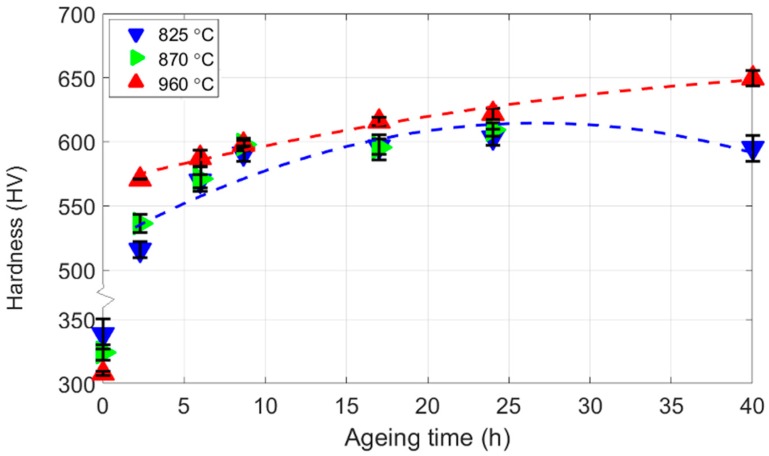
The change in Vickers hardness values with ageing time at 540 °C for the different austenitisation temperatures. The error bars show the standard deviation of the hardness measurements.

In Figure 8 are hardness test results of the three austenitisation conditions after ageing at 540 °C for a range of ageing times. After austenitisation (0 h) the lower the austenitisation temperature the higher is the hardness; 825 °C has a hardness ~30 Vickers Pyramid Numbers (HV) greater than 960 °C. After ageing for 1 h the hardness increases by around 80%, and now the higher austenitisation temperatures give higher hardness. With further ageing the hardness of all three treatments increases up to around 16 h and then falls. The difference in hardness between the treatments falls with ageing time up to around 20 h, so that at 16 h the difference has fallen to 6 HV. At longer ageing times the difference between hardness, of the high and low austenitisation conditions, increases. The hardness of 825 °C falls between 24 h and 40 h, whereas the hardness of 960 °C continues to increase.

**Figure 9:**
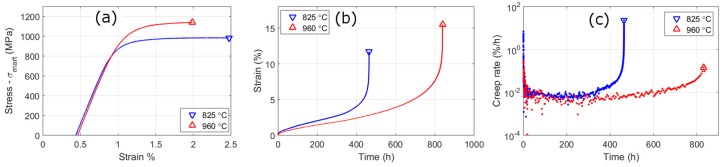
(**a**) Room-temperature tensile stress-strain curves (where *σ_mart_* is the predicted martensite strengthening); (**b**) creep-curves to rupture and (**c**) creep-rate curves. Creep samples are measured under constant stress conditions at 500 °C with a stress of 0.65 of the 500 °C yield strength. The alloy has been aged at 540 °C for 7.5 h after the low (red) and high (blue) austenitisation treatments.

In Figure 9a are room-temperature tensile stress-strain curves of the high and low austenitisation conditions after ageing for 7.5 h. The strength is ~85 MPa higher for the high austenitisation condition, but has a slightly lower elongation to failure 2.0% in comparison to 2.5%. In Figure 9b, are creep curves of the high and low austenitisation conditions after ageing for 7.5 h, tests are carried out at 500 °C and at a stress of 0.65 of the elevated 500 °C yield strength. The creep life almost doubles for the higher austenitisation condition. The shape of the creep curves, and the creep-rate curves, are similar. The low austenitisation condition has a slightly higher amount of primary creep and a slightly higher secondary creep rate. The main difference, however, is the time at which the creep-rate dramatically increases before failure, which is twice as long for the higher austenitisation condition.

## 4. Discussion

To help understand the results presented, previous work on this alloy system is included in the discussion. In the first subsection, a model is presented and used that has shown some success in predicting the strengthening of the alloy during precipitation ageing [9]. The same parameters obtained in the previous work, for use in the strengthening equations, are used in this work. In later subsections, mechanical test results on two melts of the same alloy are discussed. The difference of composition of these alloys, relative to the one in this paper, is less than 0.1 wt % for each element. However, for each of these melts a slightly different method is used to produce the melt and the heat-treatment regime is also slightly different. The melt shown in Figures Figure 12, Figure 13, Figure 14 are from the work in [8] and will be called Melt B. In Melt B, the austenitisation temperatures were similar to this work, but the ageing was slightly different: with an ageing time of 5 h, and ageing temperatures of 540 °C for the higher austenitisation treatments (HT2 for 875 °C and HT3 for 960 °C) and 560 °C for the 825 °C austenitisation temperature (HT1). The melt discussed in Figure 14, which was used in [19] to understand the influence of the martensitic microstructure, will be called Melt C. In Melt C, the austenitisation temperature used for both heat-treatments was 960 °C and both heat-treatments were aged for 5 h at 560 °C. However, whereas HTA was quenched after this 5 h, HTB was slowly cooled over several days. In Melt C, two initial prior austenite grains (PAG) sizes were compared with GS1 having a PAG size around half of GS2.

### 4.1. SANS Analysis

The analysis of metals by small angle scattering methods is a valuable tool for metallurgists, which allows for the quantification of secondary phases or voids [9,30,31,32,33,34,35,36,37,38]. Despite the advantages of yielding bulk information, there are several difficulties with the technique, the main one arising from its non-visual nature which means that assumptions have to be made of the microstructure. For example, in the case here we have assumed a constant background, a non-changing composition of phases, a non-changing precipitate shape and distribution, scattering only occurring between precipitates and matrix (i.e., no interparticle scattering), among others. In addition, there is also more work to be done to improve the underpinning mathematics for application of the technique to metals [36,39]. The main challenge with these data is the presence of multiple contributions to the scattering: austenitisation and ageing Laves phase, NiAl phase and the martensitic microstructure. The scattering intensity at different *q* values from these contributions overlap making it difficult to separate them. In a previous work [9] we showed the difficulty in separating ageing Laves phase and NiAl, and in this work we highlight the challenge in separating the larger scatterers: austenitisation Laves phase and the martensite matrix. The scattering from the martensitic matrix gives a significant amount of scattering at low values of *q* (see Figure 5). This scattering will change based on the martensitic structure, plus it is also difficult to fully account for the thickness of the sample to normalise the intensity although, in the present case, data have been normalised to measured thickness and are presented on an absolute scale. In contrast, at low *q*-values the scattering from austenitisation Laves phase is much smaller, so that the ratio of scattering between Laves phase and martensite at low *q*-values is very small (<1:10^4^). It is this reason that a simple approach to austenitisation Laves phase and the background scattering has been adopted in this paper. There are two possible ways that could help reduce these problems: (1) additional measurements made at lower *q* values would provide greater certainty in what is martensite and what is austenitisation Laves phase scattering (the intensity from a scatterer will reach a plateau at low enough *q* value based on its size such that its diameter D ~ *q*^−1^); and (2) by using a magnetic field to separate magnetic and nuclear scattering, since the different phases will provide different contributions to both.

Even with the limitations discussed above of using SANS for characterisation of precipitates in this alloy, the data still provides a valuable way to quantify the precipitate population in this alloy. The main inferences made about the difference between precipitate population for the different austenisation heat-treatments should be unaffected by the uncertainties discussed. This observation can be understood by observation of Figure 7. This figure shows that a higher austenitisation temperature equates to a greater volume fraction of smaller precipitates (~10 nm) and a smaller fraction of large precipitates (50+ nm). The issue with the SANS measurements for this complicated alloy should be thought of as being somewhere between qualitative and quantitative measurements. An example that the data can be used in a quantitative way was shown in a previous work [9] on the alloy, where the strengthening and precipitate evolution from SANS data were shown to be consistent with expectations, models and other results.

### 4.2. Strengthening Mechanisms

The SANS results can be used to understand the mechanical properties, and the precipitate strengthening. Based on previous work [9] the following equations are used for precipitate strengthening of the alloy from Laves phase (Δ*σ_L_*) and NiAl (Δ*σ_β_*):

(1) $$\Delta \sigma_{L}=\frac{0.81M_{T}Gb\ln\frac{2r_{s}}{b}}{4\pi r_{s}\left[{\left(\frac{\pi }{4\varphi }\right)}^{\frac{1}{2}}-1\right]{\left(1-\nu \right)}^{\frac{1}{2}}},\;where\;r_{s}={\left(\frac{2}{3}\right)}^{\frac{1}{2}}r$$

(2) $$\Delta \sigma_{\beta }=\min\left(\Omega_{\beta ,Oro}|\kappa_{\beta ratio}\Omega_{\beta ,Sh}\right)$$

(3) $$\Delta \sigma_{\beta ,Oro}={\left(\varphi \right)}^{\frac{1}{2}}\frac{0.81M_{T}Gb\ln\frac{2r_{s}}{b}}{4\pi r_{s}\left[{\left(\frac{\pi }{4}\right)}^{\frac{1}{2}}-0.2\right]{\left(1-\nu \right)}^{\frac{1}{2}}}\;\Delta \sigma_{\beta ,Sh}={\left(\varphi \right)}^{\frac{1}{2}}\frac{2M_{T}S}{br{\left(\pi \omega_{q}\right)}^{\frac{1}{2}}}{\left(\frac{\gamma \omega_{r}r}{S}\right)}^{\frac{3}{2}}$$

where, *M_T_* is the Taylor factor, *G* the shear modulus of the matrix, *b* the magnitude of the Burgers vector of dislocations, *ν* the Poissons ratio, *ω_q_* and *ω_r_* are constants derived from particle statistics, *S* is the dislocation line tension given by *S* = *Gb*^2^/2, *r* the particle radius and *ϕ* the volume fraction. For NiAl we also assume there is a normal distribution of particle sizes (st. dev. = 1 nm), and the total strengthening is the sum of the contributions. This has the effect of smoothing the transition between the two mechanisms.

Based on the strength of the alloy before ageing [9] we define the strength for solid solution (Δ*σ_SS_*) and from the martensite (Δ*σ_mart_*) by the following formulae:

$$\Delta \sigma_{SS}=14.5x_{Al}^{\prime }+14.5x_{Mo}^{\prime }-4.4x_{Ni}^{\prime }+14.5x_{W}^{\prime }\;HV$$

(4) $$\sigma_{mart}=\sigma_{Fe}+\sigma_{l}=260.6\;\mathrm{HV}$$

where *x’* is the solute concentration in weight %.

The overall strength is then given by:

(5) $$\sigma_{Y}=\sigma_{mart}+\Delta \sigma_{SS}+\kappa_{L}\Delta \sigma_{Oro}+\kappa_{\beta }\Delta \sigma_{\beta }$$

*κ_L_*, *κ_β_* and *κ_βratio_* are fitting variables that are determined from fitting to SANS data at a range of ageing times and temperatures to the hardness values obtained [9]. The values used are *κ_L_* = 0.60, *κ_β_* = 0.45, *κ_βratio_* = 1.42.

**Figure 10:**
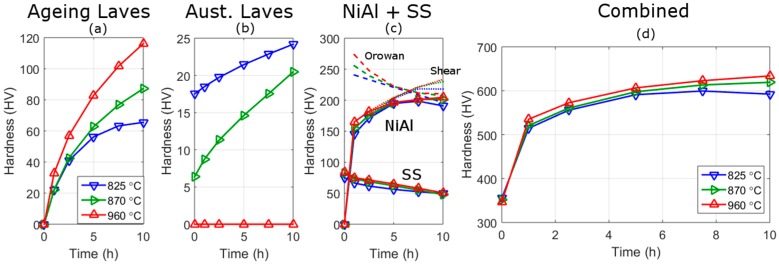
Hardness strength prediction based on the SANS analysis and mechanical strengthening Equations (1)–(5) for the ageing Laves phase (**a**); austenitisation Laves phase (**b**); NiAl phase and solid solution strengthening (SS) (**c**); and the combination of the strengthening mechanisms (**d**). The results are for the three austenitisation treatments. In the NiAl + SS plot, the NiAl curves are at the top, the full lines are the calculated strengthening, the dashed line (falling with increasing time) are from the Orowan strengthening mechanism, and the dotted line (seen increasing above calculated values at high ageing times) are from shear strengthening of NiAl precipitates.

Using these strengthening formulae and the parameters obtained from fits to the SANS data, it is possible to estimate the effect of austenitisation on the strength from different mechanisms, as shown in Figure 10; these predictions are based on polynomial fits to the volume fraction and size data (lines) shown in Figure 6.

Both the ageing and austenitisation Laves strengthening increase with ageing time but the strengthening is much greater for the ageing Laves than austenitisation Laves phase. The ageing Laves phase has a higher strengthening because of its smaller size relative to the austenitisation Laves phase. This means that the relative strengthening from ageing Laves phase increases at a greater rate, with ageing time, than the strengthening from the austenitisation Laves phase. The result of this is that after ~3 h all three austenitisation conditions have the same strength and, at longer times, the strength increases with increasing austenitisation temperature (and volume fraction of ageing Laves phase).

The strengthening from NiAl precipitates produces a characteristic increase in strength, a peak strength at ~8 h and a fall in strength. From the model this is a result of a transition from how dislocations interact with the precipitates, changing from a shear [40,41,42,43] to an Orowan mechanism [44], at a crystal diameter of approximately 4–5 nm. The size and volume fraction of NiAl is approximately the same with ageing for the three different austenitisation conditions, hence should produce approximately the same strengthening (the variation is discussed above). The solid solution strength after austenitisation and no ageing, decreases with decreasing austenite temperature; this is due to the precipitation of the Laves phase during austenitisation reducing the solid solution elements. The solid solution strengthening falls with increasing ageing times and at 10 h the strengthening is approximately the same for all austenitisation treatments.

The sum of the strengthening contributions provides the overall predicted strength with ageing time, and is shown in Figure 10. The predictions share many features of the hardness results shown in Figure 8 including: (a) a similar magnitude of hardness values; (b) a similar change in hardness with time; (c) the relative hardness of the different austenitisation temperatures.

**Figure 11:**
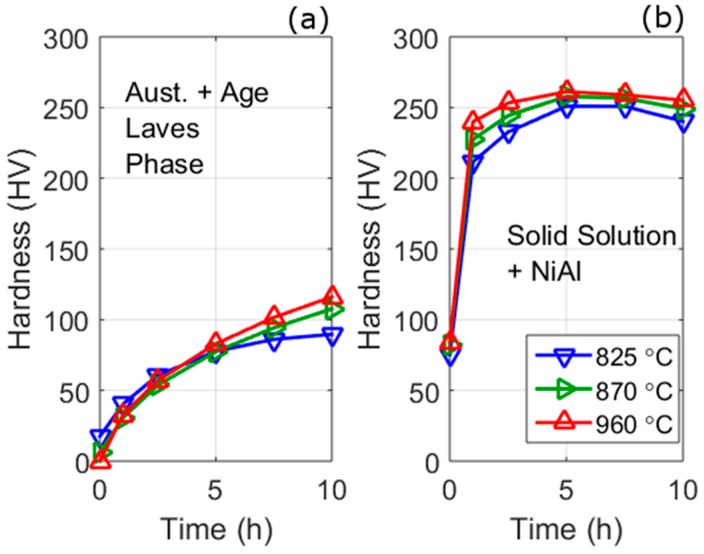
Strength predictions using SANS analysis separated based on contributions from Laves phase (**a**) and other mechanism (**b**).

To gain more information on the predictions it is worthwhile to separate the strengthening contributions into two types: strengthening from Laves phase and other strengthening mechanisms (as shown in Figure 11). The higher predicted strengthening of the 960 °C austenitisation condition at ageing times less than 5 h is caused mainly by higher strengthening from NiAl, and to a lesser extent solid solution strengthening. This strengthening is due to the higher volume fraction of NiAl seen at 960 °C (Figure 6). There are two possible cause of this increased quantity of NiAl: it may be a result of the austenitisation Laves limiting the growth of NiAl in the lower austenitisation temperatures or, alternatively, it may be because Laves precipitates are contributing to the NiAl scattered distribution, which is possible because of the underlying assumptions employed in the analysis in separating precipitates by size.

The difference between the measured hardness before ageing (~30 HV), of the high and low austenitisation conditions, is higher than that predicted by the model (~10 HV). Solid solution strengthening is predicted to be higher for the high austenitisation condition and, if we consider the strength from the austenitisation Laves phase only, the difference between predictions and measurements is closer at ~17 HV.

**Figure 12:**
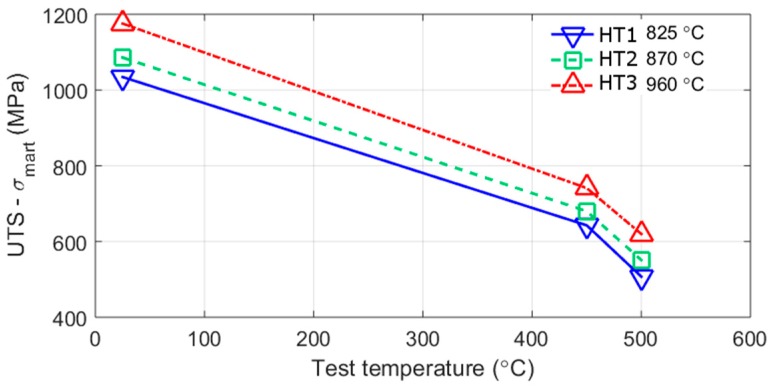
The change in ultimate tensile strength (UTS) minus the strengthening from martensite *σ_mart_* (a), of an alloy with a similar composition to that measured here and reported in [8], the alloy is called Alloy B, see start of discussion for more details. The three heat-treatments have the same austenitisation temperatures in this report and are aged for 5 h; the higher austenitisation temperatures (HT3 and HT2) are aged at 540 °C, and 825 °C at 560 °C (HT1).

After ageing the predicted and measured strengths are comparable. After 7.5 h ageing the predicted difference in hardness of 960 °C and 825 °C treatment is 23.5 HV. The difference in measured hardness between the austenitisation treatments is 18.1 HV after 6 h ageing and 6.7 HV after 8.5 h ageing, or 23.5 HV using the fitted quadratic lines. Whereas, the tensile strength difference after ageing for 7.5 h is 85 MPa which can be converted to give an approximate hardness difference of 28 HV, using HV = MPa/3.0 [45,46]. In a separate work on this alloy in Melt B, three different heat-treatments were compared, more details of the melt is given in [8] and the start of the discussion. In this work, higher austenitisation treatments were also shown to give greater strengths, as shown in Figure 12. From the figure the tensile strength is found to be 141 MPa (~47 HV) higher for the high austenitisation treatment. This is larger than that seen here but may be due to the different ageing temperatures (540 °C and 560 °C) used.

Being able to estimate creep performance is more difficult than predicting strength because the martensite and precipitates change during the course of creep; diffusional mechanisms (Coble or Nabarro-Herring creep) will occur in addition to the dislocation mechanisms. However, in the present case, where the test stress is high relative to the yield strength and the temperature low relative to the melting temperature, dislocation slip is expected to be the dominant mode of deformation. A good example of why this should be the case is the increase in creep life with reduced prior austenite and martensitic grain sizes seen at similar creep conditions in other work on this alloy [19]. The increased tensile strength and hardness of the 7.5 h aged samples austenitised at the higher temperature would therefore also be expected to perform better during creep tests. From Figure 12, on this alloy with slightly different processing, which we are calling alloy B [8], it can be seen that the increased room-temperature strength from the higher austenisation temperature (HT3 in this case), is maintained at higher temperatures. This is not always the case as shown for the effect of martensitic microstructure shown in another work on the alloy [19]. The SANS results suggest that precipitate evolution during creep would lead to lower creep strains and greater creep rupture times at the higher austenitisation conditions; this is because the strength difference between the heat-treatments increases with ageing time and thus may also be expected to increase during creep testing. The cause of this is mainly a result of the relative growth of smaller and larger Laves phase at the different austenitisation temperatures, with the smaller ones contributing more to strengthening, for the same volume fraction, than the larger ones.

**Figure 13:**
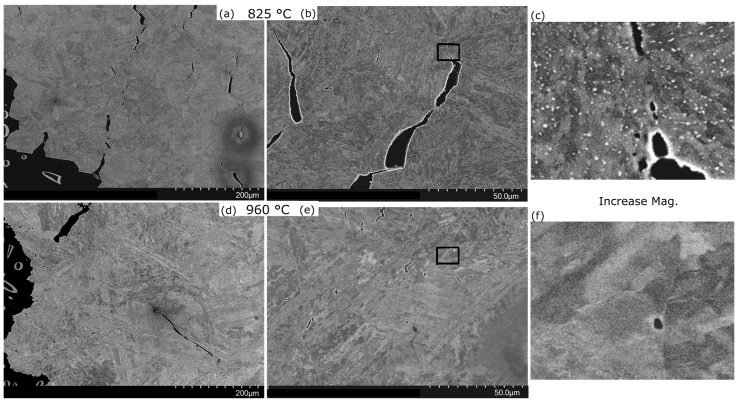
Back-scattered SEM images of failed creep samples. (**a**–**c**) after heat-treatment of 825 °C for 2 h (HT1), (**d**–**f**) after heat-treatment of 960 °C for 1 h (HT3); both are subsequently aged for 5 h. This alloy, which we call Alloy B, has a similar composition (more details may be found in [8] and at the start of the discussion section). Images are taken near the fracture surface on a plane parallel to the direction of load and perpendicular to the crack. The images on the (**c**,**f**) are taken from the middle images but with a 3 times greater magnification.

However, there is an alternative explanation. When the creep samples are viewed after failure, those austenitised at low temperatures display more cracks (Figure 13). The cracks are mainly along boundaries that have been identified from EBSD as PAG boundaries. Therefore, the presence of large Laves phase on boundaries could add strain to the creep curve and be causing earlier failure. How much this strain contributes to the creep curve is not easily determined from these tests. The creep rate curves (Figure 9c) give some justification for this being a significant factor; this is because the main separation in the creep-rate curves is the difference in the transition to tertiary creep and failure of the low austenitisation condition at lower times, rather than any substantial difference in the secondary creep-rate.

### 4.3. Failure Mechanisms

**Figure 14:**
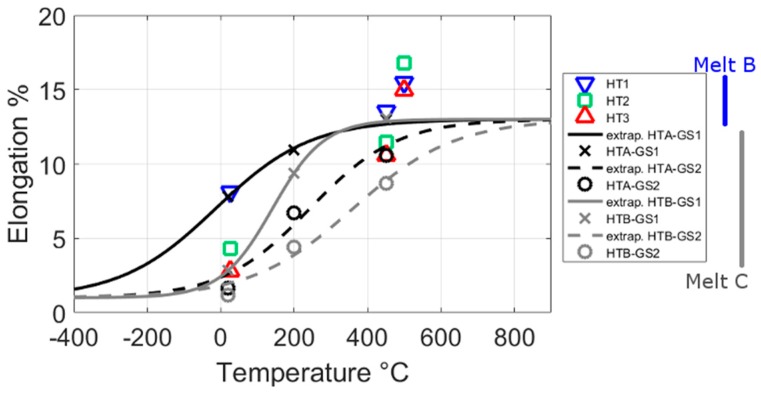
The change in elongation to failure of Melt B and Melt C, which are the same alloy as that in this paper but with slightly different processing. The three heat-treatments of Melt B have different austenitisation temperatures of 825 °C (HT1), 875 °C (HT2) and 960 °C (HT3). For more details on the melts see the start of the discussion or [8,19].

The changes in the elongation to failure after different austenitisation temperatures and at different test temperatures from the same alloy [8,19], with slightly different production and heat-treatment route, are shown in Figure 14. HT1, HT2 and HT3 are from one melt (Melt B), the same one discussed in Figure 12 and Figure 13 [8], whereas HTA and HTB are from a different melt discussed in [19] (Melt C). The results of Figure 14 show that both these alloys, and related heat-treatments, show a marked drop in elongation to failure with test temperature. In addition, this fall in elongation to failure is much more gradual in Melt B for the low austenitisation condition HT1, than the higher austenitisation condition HT3. For the larger grain size samples of Melt B (GS2), the failure occurs in a very brittle manner before the yield point. The room-temperature elongations to failure in this work (shown in Figure 9) are consistent with the previous work. That is higher austenitisation temperatures lead to a more brittle alloy when tested at room-temperature.

To understand these difference in failure and the mechanisms that may be at play, it is worth considering the changes in elongation to failure with test temperature in more detail, as shown in Figure 9. With falling test temperatures, maraging steels, and other alloys with a body centred cubic crystal structure, transition from ductile to brittle behaviour [47], at a temperature called the ductile to brittle transition temperature (DBTT). The DBTT, is due to the temperature sensitivity of the stress required to move a dislocation in body centred cubic metals and is most often used to describe failure in toughness testing but the same mechanism should be active during tensile tests. Although the name suggests the DBTT transition occurs at a single temperature this is often not the case, and in addition the temperature and length of this transition can differ depending on the test type. Figure 9 shows that there is a significant change in elongation to failure with temperature. The fracture surfaces of these samples [19] also shows a marked change from being of a brittle nature at room-temperature to being of a ductile nature at higher temperatures. In addition, impact and fracture toughness tests of this and similar alloys show a transition from ductile to brittle fracture at around room-temperature. Hence, in this alloy tensile failure at around room-temperature appears to be controlled by this ductile to brittle (DBT) effect. The DBTT can shift to higher temperatures (causing a more brittle failure at a given temperature) when the movement of dislocations is inhibited, which can occur because of precipitation or solid solution strengthening [48,49,50]. Hence for the high austenitisation condition, the small ageing Laves phase, along with NiAl and solid solution strengthening, may be inhibiting the start of dislocation motion (or yielding) so that, when plastic deformation commences, it results in an avalanche of dislocations and a more brittle failure. Large precipitates and precipitates that form along grain boundaries can act to encourage failure by crack initiation or propagation; it is this mechanism that is mostly reported for the loss in toughness of an alloy due to Laves phase [4,5,6]. The low austenitisation treatment has a greater number of larger Laves phase precipitates and precipitates forming along boundaries, and, after mechanical testing, has a greater number of cracks along grain boundaries [8]. Nevertheless, this treatment leads to a greater elongation to failure in tensile tests (Figure 9 and Figure 14). Hence, the DBT effect appears to be the dominant mechanism controlling failure, rather than from failure mechanisms from large Laves phase precipitates in this situation.

### 4.4. Martensitic Microstructure

In martensitic steels the influence of different boundary types on mechanical properties is not well understood [19] and is complicated because smaller PAGs will often lead to smaller blocks and smaller lath sizes.

The strengthening from a martensitic structure (*σ_mart_*) with a block size, *d*, can be given empirically as in Equation (6) [51,52], where *σ*_0_ is the friction stress and *k_y_* a proportionality constant taken as 684 HV μm^−1/2^ is used based on prior work on this alloy [19] and the work of Dingley and McLean [53].

(6) $$\sigma_{mart}=\sigma_{0}+k_{y}d^{-1/2}$$

Using the measured block sizes of the alloys and Equation (6), the hardness resulting from strengthening from the martensite structure plus the intrinsic strength of Fe (i.e., not accounting for solid solution or precipitation strengthening) at the different austenitisation temperatures is calculated to be: 215 HV for 825 °C, 228 HV for 870 °C, and 204 HV for 960 °C. As was discussed in a previous work [19], what contributes to the Hall-Petch grain size strengthening in a martensitic alloy remains ambiguous because there is often a relationship that exists meaning that smaller prior austenite grain sizes, leads to smaller block and lath sizes. Meaning that there is an ambiguity over the influence of the block and lath size in strengthening. In addition to this the previous work showed there is also a precipitation contribution to the grain size effect which is not well understood.

This difference in predicted strengthening of the martensitic microstructure adds to the difficulty in modelling the alloy, because it makes it difficult to separate changes in the martensitic microstructure (i.e., block and lath size), from changes in precipitation. In this work, it was shown that there was a higher predicted difference in the total strengthening after austenitisation between 825 °C and 960 °C than that measured. The prediction of 30 HV is three times higher than the measured difference of 10 HV. This discrepancy may be partly explained by the predicted strengthening due to the martensitic structure discussed above. The inclusion of the martensitic strengthening would reduce the predicted difference in the total strengthening after austenitisation between 825 °C and 960 °C to 19 HV, much closer to the measured difference of 10 HV.

A secondary effect that a smaller grain size can have on mechanical properties is to reduce the DBTT and hence increase the elongation to failure [49,54]. Hence, the changes in elongation to failure seen in Figure 14 may be a result of the martensitic microstructure and not the precipitate population. Although, the argument that failure is controlled by the DBT effect remains.

### 4.5. Characterisation of Maraging Steels

In maraging steels quantification of precipitation remains a particular problem; this problem exists both due the importance they play in the alloy’s final properties and in the difficulties in characterisation of precipitates that range from nanometres to hundreds of nanometres [9,55,56]. Although, transmission electron microscopy and atom probe tomography can provide useful information for small precipitates, the small volumes of material sampled are a limiting factor. In contrast, small angle neutron scattering experiments have in this work permitted the quantitative examination of the changes in the size and density of precipitates of a range of sizes. The technique of small angle scattering (SAS) is not unique in the ability for bulk quantification of precipitates, particularly those smaller than 50 nm; for example, such characterisation is possible by powder diffraction or positron annihilation spectroscopy [57,58,59]. However, other techniques are either not as well developed analytically, such as positron annihilation spectroscopy; or have other limitations. The latter point being a limitation in using powder diffraction (PD) for small precipitates or precipitates with a low volume fraction. When precipitates are small they are difficult to view by PD due to crystal size broadening which leads to diffraction peaks that are low in intensity and broad, and so is limited unless the source is of sufficient high resolution and intensity [60,61]. In addition, PD is unable to separate precipitates of the same crystallography, as often is the case for precipitates in the earlier stages of their growth [15,47]. SAS is a valuable tool for studying precipitation because the technique is able to gain quantifiable information on precipitation across several length scales (1 nm to 1 μm), can pick up small volume fraction changes, and is not limited by the crystallography of precipitates. SAS has many advantages over other techniques, but as discussed in this work there are still issues to be resolved. The main problems identified in this report of SAS are the presence of multiple phases and the scattering from the martensitic microstructure.

The second characterisation problem that needs to be addressed in martensitic steels is the quantification of the martensitic microstructure. EBSD is a valuable technique allowing quantification of both the prior austenite grain size and the block size (or effectively the crystal size), which would not be possible or difficult by other methods. There are limitations in the quantification of these quantities by EBSD due to the length of time to conduct a scan for a given area, because the step size needs to be reasonably small (around 1 μm or less) because of the small size of the martensitic blocks. In addition, the technique is also limited for studying the martensitic microstructure is its inability to quantify the lath size or the general disorder of the crystal lattice structure. If a small step size is used by EBSD, as done in a previous work [19], an idea of the lath size can be obtained but since it is only based on orientation changes the approach is limited. TEM is an effective way to visualise the lath and dislocation structure [8,62]. However, the method is limited by the small volume sampled, in addition to problems with quantification when the dislocation density is high [63]. Other methods such as diffraction peak profile analysis [64,65] and positron annihilation spectroscopy [65,66,67,68] have shown some success in investigating the martensitic structure. In addition, SAS technique using low *q*-values (such as USANS) may prove an effective method of quantification in the future. The use of these techniques for these applications is limited, hence further work is needed for verification of their effectiveness for this application.

## 5. Conclusions

In this work a maraging steel was subjected to a two-stage ageing heat-treatment, involving an initial austenitisation treatment at 825 °C, 870 °C or 960 °C, followed by ageing at 540 °C. Several characterisation techniques were used to quantify the microstructure during processing: small angle scattering, scanning electron microscopy, electron backscatter diffraction, and atom probe tomography. This characterisation was used to understand the change in mechanical properties from hardness testing, tensile testing and creep testing.

Martensitic steels, and in particular maraging steels, consist of multiple phases with sizes that range across several length scales, as such they represent a challenging problem within materials science. The challenge is two-fold, consisting of difficulties in characterisation of the microstructure, and difficulties relating this microstructure to mechanical properties. In this work, some of the characterisation methods available are discussed along with their advantages and limitations. It is concluded that the combination of bulk characterisation methods, EBSD and SAS with more direct techniques of SEM and APT is a powerful tool to study maraging steel. These combined techniques have limitations, but they also have several distinct advantages over other comparable techniques. As such their use should be a consideration for all researchers working on maraging steels, or alloys with small precipitates and martensitic-type microstructures.

This multiple-techniques approach has allowed for a detailed understanding of the alloy during processing, which would otherwise either not be possible or difficult. This includes:

- Quantification of precipitates from several nanometres to several 100 nanometres during processing.
  ○ It was shown that during austenitisation at the lower two temperatures, large Laves phase precipitates were precipitated on and within the prior austenite grain boundaries. As a result, the volume fraction of fine Laves precipitates that form during ageing was reduced. Although, the absolute volume fraction of Laves phase was relatively constant, the choice of austenitisation temperatures allows for the ratio of small and large Laves to be changed.
- Quantification of the martensitic microstructure of the austenitisation heat-treatments.
  ○ It was shown that the highest austenitisation temperatures had larger prior austenite grain sizes and block sizes, than the lowest.
- The changes in microstructure were related to changes in hardness, tensile and creep properties using strengthening models.
  ○ It was shown that the production of austenitisation Laves phase is detrimental to strength and creep properties, because ageing Laves have a greater contribution to strengthening. Conversely, the ageing Laves precipitates were found to be detrimental to elongation because they raise the ductile to brittle transition temperature.
  ○ The difference in martensitic microstructure from the initial austenitisation treatment will add additional strengthening to lower austenitisation treatment as well as reduce the ductile to brittle transition temperature. This highlights how the influence of a heat-treatment regime often results in changes, sometimes rather complex, on many microstructural parameters and hence mechanical properties.

## Figures and Tables

**Table 1:** The composition of the alloy studied in weight %.

C	Cr	Mo	Ni	Al	Co	W	B	Fe
0.003	10	2.75	7	1.8	8.3	2.45	0.002	Balance

**Table 2:** Precipitate area fraction and precipitate equivalent circular diameter from back-scattered SEM images and image-J analysis, data are also shown in Figure 4. The numbers in brackets are the standard deviation in the values found from the images for a particular heat-treatment condition.

Austenitisation Temperature (°C)	Area Fraction (%) after Different Ageing Times in h				Precipitate Equivalent Circular Diameter (nm) after Different Ageing Times in h			
	0	1	9	24	0	1	9	24
825	6.77 (1.23)	6.12 (1.73)	6.14 (1.14)	5.51 (1.21)	204.1 (16.8)	203.7 (16.2)	200.6 (14.4)	215.0 (20.7)
870	4.19 (1.23)	-	-	-	201.0 (16.0)	-	-	-
960	0.14 (0.05)	0.09 (0.07)	0.22 (0.10)	0.28 (0.06)	172.1 (4.5)	166.1 (18.7)	163.8 (10.6)	175.9 (24.3)

**Figure A1:**
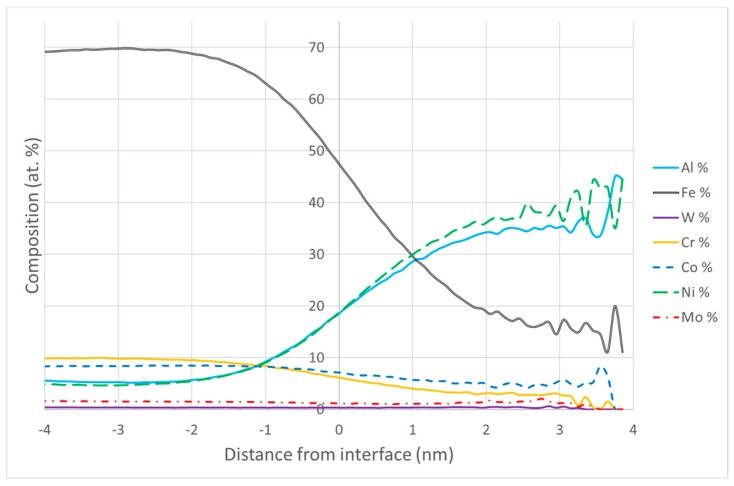
A composition proxigram of NiAl precipitates in the alloy after austenitisation at 960 °C and ageing at 540 °C for 5 h. The isosurface used to determine the proxigram was 17 atm. % Ni.

**Figure A2:**
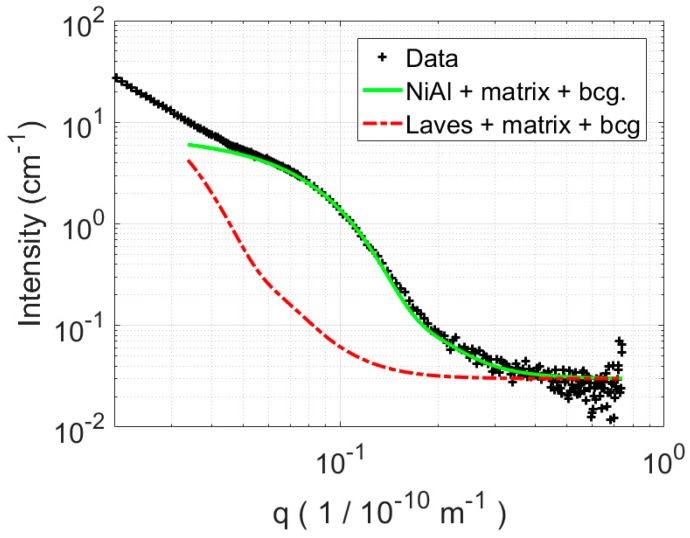
Example fit using SasView for a sample austenitised at 960 °C for 1 h and then aged at 540 °C for 1 h.

**Figure A3:**
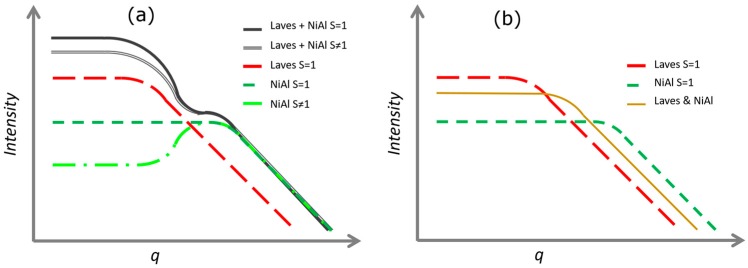
A schematic representation of possible contributions to the SANS results from interparticle scattering. In (a) it is shown how interparticle scattering between NiAl precipitates can cause a reduction in the intensity of scattering at lower q values. This would lead to an underestimation of the amount of Laves phase present; In (b) scattering between two phases can result in an additional scattering curve between the other two. The presence of this scattering could have a number of effects, but one of these is an overestimation in the volume fraction of NiAl.

## Appendix A. Small Angle Scattering Analysis

In this section, more details are provided about the methodology used for the SANS analysis.

The scattering length density between precipitate and matrix *ρ* is calculated using:

(A1) $$\rho =\frac{n_{a}}{V_{a}}\underset{i}{\sum }x_{i}b_{i}$$

where *n_a_* and *V_a_* are the number of atoms and volume of the unit cell respectively, *x_i_* is the atomic fraction of element *i* with corresponding neutron scattering length, *b_i_* [69]. We use a unit cell length of the BCC unit cell of 2.88 × 10^−10^ m for both the matrix and NiAl.

An example composition proxigram for the NiAl precipitates in the alloy using a Ni isosurface of 17 atm. % is shown in Figure A1. A proxigram is an averaged composition profile either side of a given isosurface which is a surface defined by a particular composition; more details can be found elsewhere [22]. To determine the composition of a phase, the values from the proxigram nearest the centre (larger distances) of the precipitate are used. The compositions were then used to calculate the *ρ* values of the matrix and precipitates, as shown in Table A1. The values of Δ*ρ* (i.e., neutron contrast between precipitate and matrix) are slightly different for the two ageing times shown. This difference is thought to be largely due to the uncertainty in determining the composition rather than an inherent difference between ageing times. This assessment is based on: (i) the measured variations in composition profiles for samples of the same heat-treatment; (ii) the similarity observed in the proxigrams of NiAl at different ageing times (the latter is shown in [9]); and (iii) the small size of NiAl precipitates which are comparable to the interface region between precipitate and matrix (see Figure 6) of the precipitate and means the composition of the elements do not reach a plateau at the centre of the precipitate.

**Table A1:** Scattering length density values of NiAl, found from composition of NiAl after ageing at 540 °C for 5 and 7.5 h. See Equation (A1) for details for determination of neutron scattering length densities.

*Element*		*Matrix (7.5 h)*	*NiAl (5 h)*	*NiAl (7.5 h)*
	*b_i_* (×10^−6^ Å)	*x_i_*	*x_i_*	*x_i_*
*Fe*	94.5	74.8%	15.6%	14.0%
*Cr*	36.4	11.7%	2.7%	1.4%
*Co*	24.9	8.2%	4.9%	2.7%
*Ni*	103.0	3.5%	42.0%	48.0%
*Al*	34.5	0.2%	34.2%	32.2%
*Mo*	67.2	1.0%	1.2%	1.2%
*W*	48.6	0.5%	0.5%	0.5%
*ρ* (×10^−6^ Å^−2^)		6.87	6.15	6.39
Δ*ρ* (×10^−6^ Å^−2^)			0.72	0.47

The absolute scattered intensity (*I*(*q*)) was fitted to the following function [70,71]:

(A2) $$I\left(q\right)=\underset{i}{\sum }V_{i}\varphi_{i}\Delta \rho_{i}^{2}P_{i}\left(q,r\right)S_{i}\left(q\right)+GP\left(q\right)+Bcg$$

where *ϕ* is the volume fraction of a given precipitate, given by the subscript *i*; *r* is the radius of the precipitate; *V* is the volume of the precipitate; Δ*ρ* the difference in scattering length density between the precipitate and matrix; *P*(*q,r*) is the particle form factor which, in this case, corresponds to spherical and ellipsoidal particles [70]; *S*(*q*) is the structure factor which describes scattering interference between precipitates and, here, is assumed to be equal to one (i.e., no scattering between precipitates); *GP*(*q*) is the Guinier-Porod function and is used to model the scattering from the martensitic matrix [71]; and *Bcg* is the instrument background (*Bcg* ~ 0.03 cm^−1^, and is constant with *q*).

The sizes and volume fractions of the precipitates were determined by least-square fitting of the SANS data to Equation (A2) using SasView [24]. However, the volume fractions that were obtained from this were unrealistic and so the values were multiplied by a constant to obtain values consistent with those obtained by APT at a given ageing time. This is the same as giving each precipitate its own value of Δ*ρ*. The reason for this discrepancy is discussed briefly below and in [9].

Figure A2 is an example fit of the SANS data at high *q* values. The sample has been austenitised at 960 °C and then aged at 560 °C for 1 h (the SasView fit file, the model function and datafile are included as a supplement). The sample is used to display a situation where scattering from NiAl should dominate because NiAl forms more quickly than Laves [9]. Using the Δ*ρ* value given in Table A1 for the 5 h aged sample, the volume fraction obtained for NiAl is 190% which is obviously too large. To get a more realistic volume fraction Δ*ρ* would need to be higher. For example, if Δ*ρ* were √10 times greater, or ~2.3 × 10^−6^ Å^−^^2^, by using Equation A2 the volume fraction would be a more realistic 19%. The cause of the discrepancy is not clear but is thought to arise from the following factors: (i) the presence of small precipitates of Laves and an additional Cr rich phase (both of which will have higher Δ*ρ* values than those calculated for NiAl); (ii) structure factor contributions from inter-precipitate scattering (i.e., *S*(*q*) ≠ 1); (iii) the contribution of magnetic scattering (which is not considered in the contrast determination) and (iv) intrinsic uncertainties in determining the composition of NiAl. These factors are not exhaustive but since the scattering intensity is proportional to the product of volume fraction and scattering contrast, the origin of the discrepancy is likely to arise from a combination of the above. For the Laves phase, the volume fraction obtained from the fit was more realistic. A value of 4.7% was obtained using a calculated Δ*ρ* value of 1.71 × 10^−6^ Å^−^^2^. However, the values were still larger than found by APT and so the fitted values of the volume fraction of Laves phase was also multiplied by a constant to match APT data. There will be an uncertainty in the Laves Δ*ρ* values that will result from the same factors discussed above, with an additional uncertainty resulting from quantifying the unit cell size (since it is different to the matrix and not measured here).

Interparticle scattering, or the structure factor in Equation A2, adds another level of complexity to data analysis. In cases where there is more than one precipitate type this contribution is difficult to quantify, but it could have a significant influence on the results. Previous work on alloys has shown how the interparticle scattering, between precipitates of the same type and between different precipitates can influence the scattering. For example, Briggs et al. [72] showed considerable interparticle scattering between *α*’ in a FeCrAl alloy. This interparticle scattering was modelled using a hard-sphere structure factor, which was able to explain the drop in intensity of scattering with decreasing *q*. This is shown schematically in and is represented by the curve for NiAl S ≠ 1. This fall in intensity at low *q*-values if not accounted for would lead to the volume fraction of larger precipitates to be under-estimated (i.e., for the Laves phase in the example shown and would not explain the lower calculated value of Δ*ρ* than that used (see below)). A second type of interparticle scattering is between different precipitate types. Collins, Heenan and Stone [36] considered the effect of this in a nickel-based superalloy. They showed that this could generate a scattering curve intermediate in shape between that of the scattering curves arising from the individual precipitates (as shown in Figure A3 using the example of NiAl and Laves phase). This additional scattering could lead to a number of outcomes, one of which would be an overestimation in NiAl.

Due to the problems discussed above the values of Δ*ρ* used was 4.89 × 10^−6^ Å^−2^ for Laves and 3.68 × 10^−6^ Å^−2^ for NiAl. These values were used to best match the volume fractions APT data at specific ageing times.
